# Real-life effectiveness of indacaterol/glycopyrronium/mometasone for symptomatic relief of cough after switching from inhaled corticosteroid/long-acting β_2_-agonist therapy in patients with asthma: REACH study design

**DOI:** 10.1183/23120541.00452-2022

**Published:** 2023-03-27

**Authors:** Akio Niimi, Yoshihiro Kanemitsu, Tomoko Tajiri, Kazuya Sumi, Toshiaki Mikami, Norihiko Kondo

**Affiliations:** 1 Department of Respiratory Medicine, Allergy and Clinical Immunology, Nagoya City University School of Medical Sciences, Aichi, Japan; 2 Novartis Pharma K.K., Tokyo, Japan

## Abstract

Cough is a major symptom in patients with asthma and poses a significant burden compared with other asthma symptoms. However, there are no approved treatments in Japan, developed to specifically treat cough in patients with asthma. We present the design of REACH, an 8-week real-life study, which will evaluate the efficacy of a combination of indacaterol acetate, glycopyrronium bromide and mometasone furoate (IND/GLY/MF) in asthmatic patients with cough refractory to medium-dose inhaled corticosteroid/long-acting β_2_-agonist (ICS/LABA).

Patients with asthma (age ≥20 to <80 years) with a cough visual analogue scale (VAS) ≥40 mm will be randomised 2:1:1 to receive IND/GLY/MF medium-dose 150/50/80 μg once daily or step-up to a high-dose regimen of fluticasone furoate/vilanterol trifenatate (FF/VI) 200/25 µg once daily or budesonide/formoterol fumarate (BUD/FM) 160/4.5 µg four inhalations twice daily during the 8-week treatment period. The primary objective is to demonstrate the superiority of IND/GLY/MF medium-dose over high-dose ICS/LABA in terms of cough-specific quality of life after 8 weeks. The key secondary objective is to demonstrate the superiority of IND/GLY/MF in terms of subjective assessment of cough severity.

Cough frequency (VitaloJAK cough monitor) and capsaicin cough receptor sensitivity will be evaluated in eligible patients. Cough VAS scores, fractional exhaled nitric oxide, spirometry and blood tests, and the Asthma Control Questionnaire-6, Cough and Sputum Assessment Questionnaire, and Japanese version of the Leicester Cough Questionnaire will be evaluated.

REACH will provide valuable evidence on whether a switch to IND/GLY/MF medium-dose or step-up to high-dose ICS/LABA is beneficial for patients with persistent cough despite treatment with medium-dose ICS/LABA.

## Abstract 2

**The REACH study will provide valuable evidence for physicians on whether switching to IND/GLY/MF medium-dose or step-up to high-dose ICS/LABA is beneficial for asthmatic patients with persistent cough despite treatment with medium-dose ICS/LABA** https://bit.ly/3uQlzIc

## Introduction

Patients with asthma complain of various symptoms such as cough, sputum, wheezing, chest tightness and shortness of breath, which vary over time and limit their daily activities [1]. According to asthma treatment guidelines, such as the Global Initiative for Asthma (GINA) 2022 report, apart from reducing exacerbations and improving lung function, symptom control is considered one of the important goals of asthma management [2]. Previous studies have shown that cough is a major symptom in patients with asthma and poses a greater burden compared with other symptoms [1]. An observational study conducted in Japan reported >40% of patients with asthma having residual cough symptoms despite receiving treatment from allergy or respiratory specialists [3]. Moreover, increased symptoms of cough have been associated with increased asthma severity and exacerbation rates, poor prognosis, and decreased quality of life (QoL) [4–7]. Therefore, treating cough symptoms forms an important factor in the management of asthma.

Currently, no approved drugs have been developed to specifically treat cough symptoms in patients with asthma and only a few interventional studies have focused on cough in asthma so far. Based on the available evidence, the American College of Chest Physicians recommends inhaled corticosteroids (ICS) as the first-line therapy for improvement of cough symptoms in patients with asthma [8]. In cases where there is insufficient improvement in cough symptoms despite treatment with ICS, it is recommended to increase the dose of ICS or add a leukotriene receptor antagonist (LTRA) or long-acting β_2_-agonist (LABA). However, a significant proportion of patients with asthma still suffer from persistent cough despite appropriate treatment with ICS or add-on LABA and LTRA [3]. Indeed, cough is most refractory to ICS among various asthmatic symptoms [9]; in asthmatic patients with cough, no significant improvement in capsaicin cough receptor sensitivity was observed despite 3 months of treatment with fluticasone propionate 500 μg·day^−1^ and salbutamol as-needed [10]. Further, in cough variant asthma, although airway hyperresponsiveness improved significantly, no improvement in capsaicin cough receptor sensitivity was observed even by long-term ICS treatment [11]. The current treatment options for cough symptoms refractory to existing treatments (ICS or ICS/LABA) are limited and there remains an unmet need in clinical practice [12].

Findings from a recent study have shown that tiotropium, a long-acting muscarinic antagonist (LAMA), significantly improved subjective cough visual analogue scale (VAS) scores in asthma patients with persistent cough symptoms despite treatment with ICS/LABA and capsaicin cough receptor sensitivity in the responders (who showed ≥15 mm improvement on the cough VAS) [13]. Tiotropium has been shown to improve cough VAS scores independent of its bronchodilating effect. *Ex vivo* and *in vivo* studies using animal models also suggested that the cough suppression mechanism of LAMA is unrelated to its anticholinergic activity. Birrell *et al*. [14] demonstrated that tiotropium, but not atropine and glycopyrronium, was able to modulate the cough reflex through direct or indirect inhibition of transient-potential vanilloid receptor type 1 (TRPV1), also known as the capsaicin receptor. In addition, Mutolo *et al*. [15] indicated that not only TRPV1 but also acid-sensing ion channels as well as mechanoreceptors were involved in the mechanism of cough suppression by LAMA. Glycopyrronium bromide, another LAMA, inhibited capsaicin-induced cough in healthy volunteers and was shown to reduce cough during endoscopic submucosal dissection procedures [16, 17]. These results, although not yet evaluated in asthma, indicate a promising cough-suppressing activity of inhaled glycopyrronium bromide.

In Japan, medium-dose ICS in combination with LABA is used as first-line treatment for many patients with asthma. The Japanese Society of Allergology recommends addition of LAMA/LTRA/theophylline to existing therapy or increase of ICS as the next steps for patients with poorly controlled asthma [18]. In 2020, Enerzair Breezhaler, a fixed-dose combination of indacaterol acetate, glycopyrronium bromide and mometasone furoate (IND/GLY/MF), was approved as maintenance treatment of asthma in patients inadequately controlled on high-dose ICS/LABA in European countries, and in Japan for the treatment of bronchial asthma in patients requiring combination of ICS, LABA and LAMA [19].

A network meta-analysis that compared the benefit of add-on LAMA *versus* increased doses of ICS reported that addition of LAMA was more effective in improving lung function, while increasing doses of ICS were more effective in reducing asthma exacerbations [20]. However, the clinical question regarding the best option for residual symptoms, the most important attribute for patients with asthma using inhaler medications [21], still remains unresolved due to the lack of evidence directly comparing the effectiveness among these step-up options. Thus, in this REACH (Real-life effectiveness of Enerzair on Asthmatic CougH) study, we will address the clinical question of whether once-daily IND/GLY/MF medium-dose (150/50/80 μg) is superior to high-dose ICS/LABA for effectiveness against the most burdensome symptom (cough) in patients with asthma [1]. In this study, two different ICS/LABA were selected as the comparators: fluticasone furoate/vilanterol trifenatate (FF/VI; 200/25 µg once daily) and budesonide/formoterol fumarate dihydrate (BUD/FM; four inhalations of 160/4.5 µg twice daily). We hypothesise that IND/GLY/MF, due to its LAMA component glycopyrronium, exhibits a superior effect on improving cough symptoms than high-dose ICS/LABA in its anticholinergic activity-independent manner as described before. Therefore, we will conduct capsaicin cough receptor sensitivity testing in a subgroup of patients in order to confirm its novel mechanism of action.

### Objectives

The primary objective of the study is to demonstrate the superiority of IND/GLY/MF medium-dose over high-dose ICS/LABA (FF/VI or BUD/FM) in terms of improvement in cough-specific quality of life after 8 weeks of treatment in asthma patients with cough refractory to medium-dose ICS/LABA. The key secondary objective is to demonstrate the superiority of IND/GLY/MF medium-dose over high-dose ICS/LABA after 8 weeks of treatment in terms of subjective assessment of cough severity. The other secondary objectives are to evaluate the efficacy of IND/GLY/MF medium-dose to comparator high-dose ICS/LABA in terms of the end-points listed in table 1.

**TABLE 1:** Objectives and end-points

**Objective**	**End-point**	**Time-point**
**Primary objective**		
To demonstrate the superiority of IND/GLY/MF medium-dose over high-dose ICS/LABA (FF/VI or BUD/FM) in terms of cough-specific quality of life after 8 weeks of treatment in asthma patients with cough refractory to medium-dose ICS/LABA	Change from baseline in J-LCQ score	Week 8
**Secondary objectives**		
To demonstrate the superiority of IND/GLY/MF medium-dose over high-dose ICS/LABA after 8 weeks of treatment in terms of subjective assessment of cough severity	Change from baseline in cough severity VAS score	Week 8
To evaluate the efficacy of IND/GLY/MF medium-dose to high-dose ICS/LABA on the secondary end-points listed	Change from baseline in J-LCQ score	Week 4
	Percentage of patients achieving the MCID of ≥1.3 from baseline in J-LCQ	Weeks 4 and 8
	Cough severity VAS score during daytime (awake) and night-time (sleeping)	Weeks 4 and 8
	Percentage of patients achieving ≥15 mm improvement from baseline or absolute value of <40 mm in cough VAS	Weeks 4 and 8
	Capsaicin cough receptor sensitivity^#^	Week 8
	Cough frequency using VitaloJAK cough monitor^#^	Week 8
	FEV_1_	Week 8
	FVC	Week 8
	FEF_25–75%_	Week 8
	*F*_ENO_	Week 8
	Biomarkers (blood eosinophils and blood neutrophils)	Weeks 4 and 8
	ACQ-6	Weeks 4 and 8
	CASA-Q	Weeks 4 and 8

IND/GLY/MF: indacaterol acetate/glycopyrronium bromide/mometasone furoate; ICS: inhaled corticosteroid; LABA: long-acting β_2_-agonist; FF/VI: fluticasone furoate/vilanterol trifenatate; BUD/FM: budesonide/formoterol fumarate; J-LCQ: Japanese version of the Leicester Cough Questionnaire; VAS: visual analogue scale; MCID: minimal clinically important difference; FEV_1_: forced expiratory volume in 1 s; FVC: forced vital capacity; FEF_25–75%_: forced expiratory flow at 25−75% of FVC exhalation; *F*_ENO_: fractional exhaled nitric oxide; ACQ-6: Asthma Control Questionnaire-6; CASA-Q: Cough and Sputum Assessment Questionnaire. ^#^: capsaicin cough receptor sensitivity assessment and VitaloJAK cough monitoring would be conducted only in a subgroup of patients.

## Methods

### Study design

**FIGURE 1:**
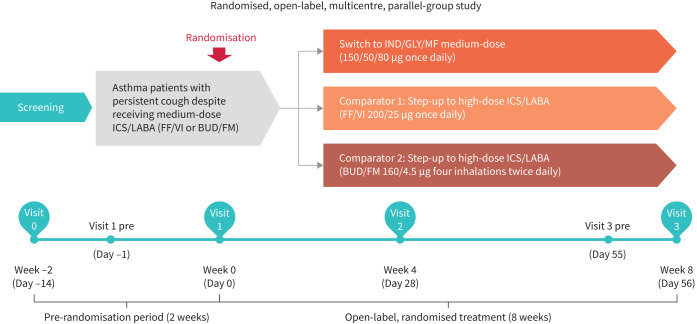
Study design. ICS: inhaled corticosteroid; LABA: long-acting β_2_-agonist; FF/VI: fluticasone furoate/vilanterol trifenatate; BUD/FM: budesonide/formoterol fumarate; IND/GLY/MF: indacaterol acetate/glycopyrronium bromide/mometasone furoate.

This is an 8-week, randomised, open-label, multicentre, parallel-group study in a real-life setting. All patients must have received medium-dose ICS/LABA therapy with FF/VI or BUD/FM for at least 1 month at stable doses prior to the screening visit. Following a 2-week screening period, at the start of the treatment period (Day 0), eligible patients will be randomised in a 2:1:1 manner to one of the three treatment arms: IND/GLY/MF medium-dose 150/50/80 µg once daily (Enerzair Breezhaler; Novartis, Basel, Switzerland) or step-up to a high-dose regimen from the current treatment of FF/VI 200/25 µg once daily (Relvar 200 Ellipta; GlaxoSmithKline, London, UK) or BUD/FM 160/4.5 µg four inhalations twice daily (Symbicort Turbuhaler; AstraZeneca, Cambridge, UK) during the 8-week treatment period (figure 1). In the BUD/FM group, dose reduction to three inhalations twice daily is allowed, only if any safety concern arises with the increase in dose of formoterol. All patients will be treated in an outpatient setting for 8 weeks. Prior to the start of treatment, all patients should also have a cough severity VAS score during daytime (awake) measured on both days of screening and treatment initiation (or 1 day before the start of treatment), which must be ≥40 mm on both days. The eligibility of the patients will be assessed as per the inclusion/exclusion criteria and only those fulfilling the criteria will enter the drug treatment period.

### Ethics approval and informed consent

This study will be conducted at 14 centres in Japan. At the screening visit, written informed consent will be obtained from the subjects. The study will be conducted in compliance with the ethical principles stipulated in the Declaration of Helsinki, the Clinical Research Act and related notifications, and the research protocol. The study would be approved by the Nagoya City University Institutional Review Board. Key study information (*e.g.* study design and recruitment information) is registered at the Japan Registry of Clinical Trials with identifier number jRCTs041220003.

### Study participants

The study aims to enrol approximately 212 male and female asthmatic patients with persistent cough, which persisted for 4 weeks prior to screening and during the 2-week pre-randomisation period (cough VAS ≥40 mm at Visit 0 and Visit 1), despite treatment with medium-dose ICS/LABA (FF/VI or BUD/FM) at stable doses prior to randomisation.

#### Inclusion criteria

Patients eligible for inclusion in this study should meet all of the following criteria. 1) Male or female patients aged ≥20 to <80 years at the time of informed consent. 2) Documented diagnosis of asthma for at least 3 months (with proof of diagnosis documented in the medical records) prior to the screening visit at an institution with a pulmonologist. 3) Treatment with medium doses of FF/VI or BUD/FM for at least 1.5 months prior to randomisation. 4) Cough VAS ≥40 mm at both screening and start of treatment (or Visit 1 pre). 5) Unwilling to receive single maintenance and reliever therapy (SMART) during the study period.

#### Exclusion criteria

Patients who meet any of the following criteria will not be eligible for participation in this study. 1) Patients who have smoked (including e-cigarettes) within 12 months prior to screening or who have smoked ≥10 pack-years. 2) Use of any central antitussive (*e.g.* codeine phosphate, *etc.*) or antitussive herbal medications within 1 month prior to screening. 3) Use of neuromodulators (*e.g.* opioids, gabapentin, pregabalin, amitriptyline, *etc.*) for cough within 3 months prior to screening. 4) Use of an angiotensin-converting enzyme inhibitor within 3 months prior to screening. 5) Use of anticholinergic drugs (LAMA, short-acting muscarinic antagonist and oral drugs) and tricyclic antidepressants with anticholinergic effects within 3 months prior to screening. 6) Initiation or change of chronic asthma medications within 3 months prior to Visit 0, with the exception of medium-dose ICS/LABA. 7) SMART within 3 months prior to screening. 8) Infection of the upper or lower respiratory tract, or significant change in pulmonary function within 1 month before screening or from screening until the start day of treatment. 9) Chest radiograph obtained within 12 months prior to screening with abnormal findings that may be associated with cough. 10) History of chronic lung diseases other than asthma (these include, but are not limited to, COPD, sarcoidosis, interstitial lung disease, cystic fibrosis, bronchiectasis and active infections such as pulmonary tuberculosis). 11) Patients with narrow-angle glaucoma. 12) Patients with dysuria due to benign prostatic hyperplasia. 13) Active malignancy. 14) Patients unable or unwilling to use the ePatient Diary device. 15) Participation in other interventional studies (including clinical trials). 16) Pregnant, nursing or possibly pregnant women. 17) Patients who have previously experienced an event of safety concern after administration of the study drug and each active ingredient thereof. 18) Other patients judged inappropriate as study participants by the investigator or subinvestigator.

### Randomisation

The investigator ensures that all patients who have signed the informed consent form meet all the inclusion criteria. Thereafter, patient information is entered into the electronic data capture system by trained personnel. At the start of the treatment period (Day 0), all patients who meet the eligibility criteria will be randomised (2:1:1) to one of the three treatment arms using a permuted block method stratified by gender, types of ICS/LABA products at screening and cough severity VAS score.

### End-points

The primary end-point of the study is change from baseline in the Japanese version of the Leicester Cough Questionnaire (J-LCQ) [22] score after 8 weeks of treatment with IND/GLY/MF medium-dose *versus* high-dose ICS/LABA (FF/VI or BUD/FM) in asthma patients with cough refractory to the same corresponding medium-dose ICS/LABA. The LCQ is a 19-item questionnaire comprising three health domains: physical, psychological and social [23]. The minimal clinically important difference (MCID) is 1.3 in chronic cough [24].

The key secondary end-point is change from baseline in cough severity VAS score with IND/GLY/MF medium-dose *versus* high-dose ICS/LABA (FF/VI or BUD/FM) after 8 weeks of treatment. The other secondary end-points are J-LCQ at Week 4 and cough severity VAS score during daytime (awake) and night-time (sleeping) at Weeks 4 and 8; cough receptor sensitivity at Week 8, cough frequency at Week 8, forced expiratory volume in 1 s (FEV_1_), forced vital capacity (FVC), forced expiratory flow at 25–75% of FVC exhalation (FEF_25–75%_) and fractional exhaled nitric oxide (*F*_ENO_) at Week 8; biomarkers (blood eosinophils and blood neutrophils), Asthma Control Questionnaire-6 (ACQ-6), and Cough and Sputum Assessment Questionnaire (CASA-Q) at Weeks 4 and 8.

The percentage of patients achieving the MCID of ≥1.3 in J-LCQ scores from baseline at Weeks 4 and 8 and the proportion of patients achieving ≥15 mm improvement of cough VAS score from baseline [25], or those who had an absolute cough VAS value of <40 mm at Week 4 or 8 will be also evaluated. Safety assessment will include monitoring of all adverse events (AEs), serious AEs (SAEs), unexpected SAEs and adverse drug reactions. An independent external committee to assess efficacy and safety will not be established for this study.

### Study visits and study assessments

During the study period, patients will attend at least a total of four study visits. Patients undergoing VitaloJAK cough monitor measurement would require an additional two visits (on Day −1 and Day 55) (table 2). A patient diary recording device will be provided at the start of treatment (Day 0) (for those undergoing VitaloJAK cough monitoring, the patient diary will be provided at Day −1). Adherence and daily number of doses of investigational product administered will be tracked using an electronic patient diary. Cough frequency would be measured using the VitaloJAK cough monitor only in a subgroup of patients and such eligible patients will be required to wear the VitaloJAK cough monitor. The subgroup of patients undergoing VitaloJAK cough monitor measurement would additionally undergo capsaicin cough receptor sensitivity testing, both of which would be performed on the start day of treatment and at Week 8.

**TABLE 2:** Study assessments

	**Visit 0:** **eligibility confirmation**	**Visit 1 pre:** **day −1 of administration**	**Visit 1:** **start of treatment**	**Visit 2**	**Visit 3 pre:** **day −1 of completion**	**Visit 3:** **end of treatment**	**At discontinuation**
**Week**	−2		0	4		8	
**Day**	−14	−1	0	28±7	55	56±7	
**Inclusion/exclusion criteria**	X	X	X				
**Informed consent**	X						
**Registration/allocation**			X				
**Age**	X						
**Height and weight**			X				
**BMI**			X				
**Smoking history**	X						
**Complications**	X						
**Medical history**	X						
**Duration of asthma**			X				
**Severity of asthma**			X				
**History of childhood asthma**			X				
**Family history of asthma**			X				
**Cough VAS^#^**	X		X	X		X	
**Capsaicin cough receptor sensitivity test^¶^**			X			X	
**VitaloJAK cough monitor^¶^**		X			X		
**Spirometry**			X			X	
***F*_ENO_**			X			X	
**Blood tests (eosinophils and neutrophils)**			X	X		X	
**ACQ-6**			X	X		X	
**CASA-Q**			X	X		X	
**J-LCQ**			X	X		X	
**Adverse event survey**	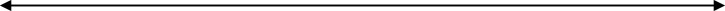						
**Survey of concomitant drugs and therapies**	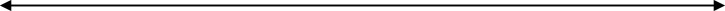						
**Use of study drug**			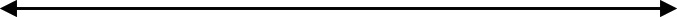				
**Status of compliance with study treatment**			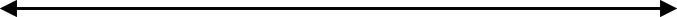				

BMI: body mass index; VAS: visual analogue scale; *F*_ENO_: fractional exhaled nitric oxide; ACQ-6: Asthma Control Questionnaire-6; CASA-Q: Cough and Sputum Assessment Questionnaire; J-LCQ: Japanese version of the Leicester Cough Questionnaire. ^#^: cough symptoms while awake and while sleeping will be evaluated using a cough VAS: “while awake” is defined as the time between 06:00 and 22:00 on the day before the examination, and “while sleeping” is defined as the time between 22:00 and 06:00 on the day of the examination, and the subjects will answer the questions at each visit using the cough VAS assessment sheet. If there is a Visit 1 pre or Visit 3 pre, it can be performed at that visit. ^¶^: performed only for patients who are eligible for the VitaloJAK cough monitor.

Cough severity VAS scores are assessed at screening and at the start of treatment while awake, and at Week 4 and at the end of study treatment (Week 8) while awake and sleeping. Spirometry measurements and *F*_ENO_ will be evaluated on the start day of treatment and at Week 8. ACQ-6, CASA-Q and J-LCQ questionnaires will be administered to the subjects on the start day of treatment and at Weeks 4 and 8 (end of treatment period). Blood samples (eosinophils and neutrophils) will be collected at the start of treatment and at Weeks 4 and 8. The AE survey will be performed from the day of informed consent until the end of treatment period.

### Statistical methods

The efficacy analysis will be performed in both the full analysis set (FAS) and the per-protocol set (PPS). The FAS will comprise subjects who received at least one dose of the study drug and have had at least one evaluable post-treatment efficacy data. The PPS will include all patients in the FAS excluding those with major protocol deviations and inclusion/exclusion criteria violations. Safety analyses of the treatments will be evaluated in the safety analysis population, which includes subjects who will receive at least one dose of study drug.

The statistical analysis will apply the following three steps, and will employ a closed testing procedure and the Bonferroni method to control the family-wise type I error at the one-sided α level of 0.025:

1) Compare IND/GLY/MF with (FF/VI, BUD/FM) for J-LCQ
2a) Compare IND/GLY/MF with (FF/VI, BUD/FM) for cough VAS
2b) Compare IND/GLY/MF with FF/VI and BUD/FM for J-LCQ
3) Compare IND/GLY/MF with FF/VI and BUD/FM for cough VAS

**FIGURE 2:**
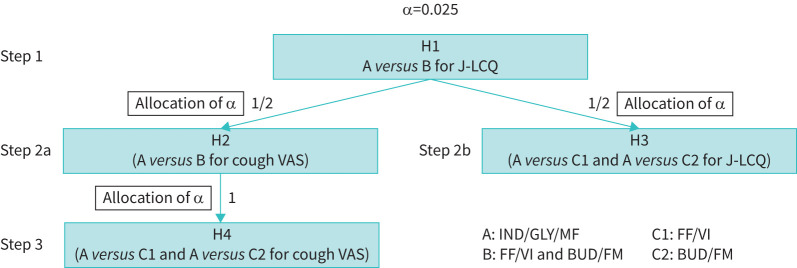
Illustration of the statistical hypothesis. J-LCQ: Japanese version of the Leicester Cough Questionnaire; VAS: visual analogue scale; IND/GLY/MF: indacaterol acetate/glycopyrronium bromide/mometasone furoate; FF/VI: fluticasone furoate/vilanterol trifenatate; BUD/FM: budesonide/formoterol fumarate hydrate.

Step 1 will employ Welch's test and IND/GLY/MF will be considered superior to (FF/VI, BUD/FM) if p<0.025, then move onto Steps 2a and 2b. Steps 2a and 2b will employ Welch's test and Dunnett's test including IND/GLY/MF as control for cough VAS and J-LCQ, respectively, and IND/GLY/MF will be considered superior to (FF/VI, BUD/FM) for cough VAS if p<0.0125, then move onto Step 3. Step 3 will employ Dunnett's test including IND/GLY/MF as control for cough VAS (figure 2).

For other secondary end-points, Welch's test and the Chi-squared test will be performed to compare the mean change from baseline between the groups and to compare the proportion between the groups, respectively. The significance level of 5% as two-sided is applied for statistical testing. No multiplicity adjustment will be performed. Missing data will not be imputed with estimates or calculations.

### Sample size considerations

To demonstrate the superiority of IND/GLY/MF medium-dose over high-dose ICS/LABA (FF/VI or BUD/FM) in terms of mean change from baseline in J-LCQ score (MCID±sd of at least 1.3±1.8) as the primary end-point and mean change from baseline in cough VAS score (MCID±sd of at least 15±20 mm) as the key secondary end-point [13, 26], following 8 weeks of treatment, with at least 80% power on a two-sided test at 2.5% level of significance and assuming a 10% dropout rate, the target sample size is set at 212 subjects. The sample size and power calculations are performed in RStudio version 1.1.456 with the MKpower package.

## Discussion

This randomised, multicentre, open-label study intends to demonstrate the effectiveness of the switch to IND/GLY/MF medium-dose in asthma patients with cough refractory to medium-dose ICS/LABA in a real-life setting. The study aims to demonstrate the superiority of once-daily IND/GLY/MF medium-dose over high-dose ICS/LABA (FF/VI or BUD/FM) as measured by the J-LCQ after 8 weeks of treatment.

The GINA 2022 report recommends the addition of a LAMA to medium- or high-dose ICS/LABA, for patients at GINA Step 5, irrespective of the reliever therapy. The LABA/LAMA/ICS combination is recommended to treat patients with asthma who are inadequately controlled and continue to experience symptoms despite treatment with medium- or high-dose ICS/LABA [2]. Previous studies suggest that the addition of LAMA to ICS/LABA improves lung function but not symptoms compared with dual therapy [27], whereas increasing the dose of ICS is effective in preventing exacerbations in asthma [20]. Therefore, the clinical question on which option is best remains, as there is no evidence comparing the effectiveness among these step-up options against the residual cough symptoms in patients with asthma. In the secondary analyses of the IRIDIUM study, treatment with once-daily IND/GLY/MF medium-dose was shown to provide greater improvements in lung function and asthma control *versus* high-dose fluticasone/salmeterol at Week 26 in patients with asthma inadequately controlled on medium- or high-dose ICS/LABA [19]. The improvements in lung function (trough FEV_1_) were rapid and were observed as early as 5 min after the administration of the first dose. These improvements in lung function and asthma control observed at Week 26 were maintained at Week 52. IND/GLY/MF was well tolerated and demonstrated a favourable safety profile [19].

In our study, the primary outcome measure is the J-LCQ evaluated after 8 weeks of treatment. The LCQ consists of 19 questions covering three health domains (physical, psychological and social) to evaluate the effect of cough on quality of life [23]. The LCQ is a well-validated tool with very good internal reliability, repeatability and responsiveness [28]. Previous studies demonstrated a significant correlation between the J-LCQ and subjective cough severity and frequency [29]. The key secondary end-point, the cough severity VAS, is the most appropriate tool [30, 31] to evaluate subjective cough severity and is the commonly used index in studies to evaluate the effect of drugs on cough. Changes in capsaicin cough receptor sensitivity and objective measures of cough frequency (using the VitaloJAK cough monitor), commonly used indicators in clinical trials to evaluate the efficacy of drugs for chronic cough, will also be evaluated in this study. Cough reflex sensitivity assessment is reproducible and responsive in patients. A recent study has shown that increased capsaicin cough receptor sensitivity is a risk factor for severe asthma and is associated with worse asthma outcomes [22]. This heightened capsaicin cough receptor sensitivity is an independent factor of daytime asthmatic cough that is refractory to ICS [32]. Nocturnal cough frequency, which can be detected using the cough monitor, may provide unique and valuable information for early prediction of treatment effect in asthma [33]. The other important parameters such as pulmonary function, *F*_ENO_ and asthma control, which are commonly evaluated in routine clinical practice, will also be evaluated in the study.

The current study uses a randomised, parallel-group design, which allows to optimise study rigour and reduce allocation bias, whereas the real-world setting contributes to increased external validity of the results. Since the study uses a real-world setting, adherence to treatments may be low compared with randomised controlled trials, which would anyway be reflective of real-world clinical scenarios. While considering the feasibility of conducting this study as part of routine clinical practice, blinding will not be performed.

Asthma patients in clinical trials are not representative of the real-life setting because of the stringent inclusion and exclusion criteria. This pragmatic clinical study does not require airway reversibility and a baseline FEV_1_ below a certain level for inclusion in the study, unlike previous clinical trials involving triple therapy such as IRIDIUM [19] and CAPTAIN [34], as the frequency of cough does not correlate with airflow limitation. These selection criteria exclude most patients from typical clinical trials [35].

Given the known pharmacodynamic properties of each component of the fixed-dose combination and the precedent of other LAMA used to treat asthma [13], the 8-week treatment period is considered to be the optimal period to test for an improvement in the primary end-point. The study design does not include a placebo control and instead uses active comparators, as it is considered unethical to use placebo in patients with symptomatic asthma. It should be also noted that such active-controlled superiority studies may require larger sample sizes than placebo-controlled trials. The current study is sufficiently powered to evaluate the superiority of IND/GLY/MF over FF/VI or BUD/FM in asthma patients with persistent cough. In addition, the selected active controls are ICS/LABA, most prevalent at least in Japan (and other developed countries such as European Union nations and the USA), representing the real-life scenario. Considering the impact of significant differences in inhalation techniques on adherence, metered-dose pressurised inhalers such as fluticasone propionate/formoterol are excluded as a comparator in this study. To the best of our knowledge, this is the first study to switch directly from FF/VI or BUD/FM to IND/GLY/MF.

In conclusion, this study is well designed to answer the clinical question on whether a switch to IND/GLY/MF medium-dose or step-up to a high-dose regimen of FF/VI once daily or BUD/FM four inhalations twice daily is more beneficial for patients with persistent cough despite treatment with medium-dose ICS/LABA.
